# Contradictions in clinical teachers’ engagement in educational development: an activity theory analysis

**DOI:** 10.1007/s10459-018-9853-y

**Published:** 2018-10-03

**Authors:** Agnes Elmberger, Erik Björck, Matilda Liljedahl, Juha Nieminen, Klara Bolander Laksov

**Affiliations:** 1 Department of Learning, Informatics, Management and Ethics, Karolinska Institutet, Tomtebodavägen 18A, 171 77 Stockholm, Sweden; 2 Department of Molecular Medicine and Surgery, Karolinska Institutet, Stockholm, Sweden; 3 Department of Clinical Genetics, Karolinska University Hospital, Stockholm, Sweden; 4 Primary Health Care Unit, Institute of Medicine, The Sahlgrenska Academy, University of Gothenburg, Gothenburg, Sweden; 5 Department of Education, Stockholm University, Stockholm, Sweden

**Keywords:** Activity theory, Clinical teacher, Contradictions, Educational development, Faculty development, Health personnel, Undergraduate medical education, Qualitative research

## Abstract

Many medical universities offer educational development activities to support clinical teachers in their teaching role. Research has focused on the scope and effectiveness of such activities and on why individual teachers attend. However, systemic perspectives that go beyond a focus on individual participants are scarce in the existing literature. Employing activity theory, we explored how clinical teachers’ engagement in educational development was affected by the systems they act within. Three focus groups were held with clinical teachers from different professions. A thematic analysis was used to map the contradictions between the systems that the participants were part of and the manifestations of these contradictions in the system of education. In our model, clinical teachers were part of three activity systems directed by the objects of patient care, research and education respectively. Contradictions arose between these systems as their objects were not aligned. This manifested through the enacted values of the academic hospital, difficulties establishing educational discussions in the clinical workplace, the transient nature of educational employments, and impediments to developing a teacher identity. These findings offer insights into the complexities of engaging in educational development as clinical teachers’ priorities interact with the practices and values of the academic hospital, suggesting that attention needs to shift from individual teachers to developing the systems in which they work.

## Introduction

Due to recurrent calls to professionalise teaching practice within health professions education (HPE) (Mclean et al. 2008), many medical universities offer educational development activities to support clinicians in their teaching role. Research has focused on the arrangement, scope and effectiveness of such activities (De Rijdt et al. 2013; Leslie et al. 2013; Steinert et al. 2016; Stes et al. 2010), as well as the reasons why individual clinical teachers participate in these programs (Steinert et al. 2010). However, not enough is known about systemic influences on clinical teachers’ engagement in educational development.

Educational development describes “actions, planned and undertaken by faculty members themselves or by others working with faculty, aimed at enhancing teaching” (Amundsen and Wilson 2012, p. 90). Previous research into higher education teachers’ participation in such educational development has shown the importance of both intrinsic factors and professional motives (Knight et al. 2006). Also, there have been efforts to investigate barriers to and opportunities for educational development at individual, group and institutional levels among medical educators (Stenfors-Hayes et al. 2010).

Focusing on teachers situated in clinical environments, the current literature offers insights into time-related barriers and individual motivation factors to participate in educational development, including personal and professional development and opportunities to meet like-minded colleagues (Sorinola et al. 2013; Steinert et al. 2009, 2010; Zibrowski et al. 2008). However, the emphasis has primarily been on individual clinical teachers, while it would be valuable to include a systemic perspective on their engagement in educational development, recognising the complex clinical environment of which they are part (Amundsen and Wilson 2012; O’Sullivan and Irby 2011; Steinert et al. 2006).

One theory that pays attention to interactions between the individual and the system is activity theory (Engeström et al. 1999; Engeström 1987; Virkkunen and Newnham 2013). It has been proposed as a tool for studying complex issues in HPE (Varpio et al. 2017) and has previously been applied to address different aspects of workplace learning (Reid et al. 2015; de Feijter et al. 2011; Larsen et al. 2017) and group interactions (Kent et al. 2016).

**Fig. 1:**
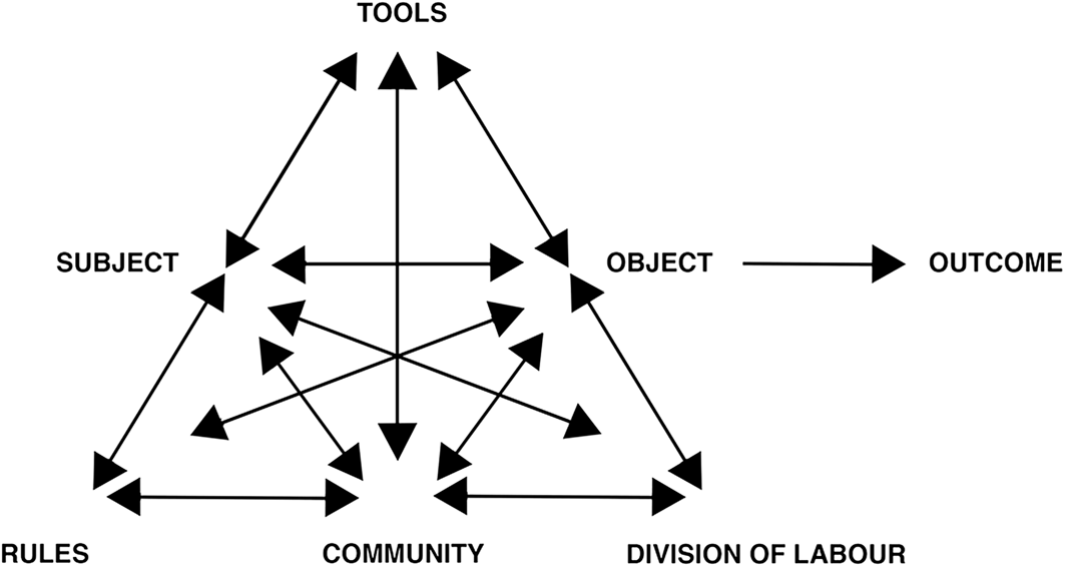
The activity system and its elements (Engeström and Sannino 2010). The subject is working on an object, the entity at which the activity is directed. The subject uses tools to mediate the actions and the activity ultimately leads to outcomes. The subject is member of a community with a division of labour among the members of the community and rules regulating the activity

The activity system (Fig. 1) is seen as the basic unit of analysis. It depicts the object-oriented nature of human activity with a subject, i.e. the individual or group whose viewpoint is chosen as the perspective of analysis, working on an object resulting in outcomes (Engeström and Sannino 2010; Leont’ev 1978; Vygotskij and Cole 1978). While the object of an activity is the motive directing it, e.g. patient care, the outcome is the product of the activity, e g. improved health. The object can be present at the individual level where it is connected to personal motivation, called ‘the specific object’, or at the system level connected to societal meaning, referred to as ‘the generalized object’ (Engeström and Sannino 2010).

The activity system also depicts the use of mediating tools for the interaction between subject and object, or in other words, how tools mediate the subject’s work towards achieving the object (Engeström and Sannino 2010; Leont’ev 1978; Vygotskij and Cole 1978). Such tools originate from our cultural history and social environment and can be concrete, such as a schedule, or abstract, such as language and concepts (Nicolini 2012). The subject is part of a social and cultural community in which rules regulate the activity and where a division of labour specifies how work is divided within the community. All elements interconnect and together mediate, or hinder, the subject’s ability to achieve the object (Engeström and Sannino 2010).

Activity systems comprise a multiplicity of views, traditions and cultures which, when misaligned, give rise to contradictions within and between neighbouring activity systems (Engeström 1987). Contradictions are historic and systemic tensions, which manifest as disturbances in the activity. They are challenging to capture in empirical studies, however, their manifestations can be recognised in individuals’ accounts and constructs of words and actions (Engeström and Sannino 2011).

Activity theory affords a tool for understanding complex interactions between individuals and systems and can aid in addressing parts of the systemness of engaging in educational development. Thus, the aim of this study was to employ an activity theory perspective to explore how clinical teachers’ engagement in educational development is affected by the systems they act within. Our specific research questions were: (1) what systems are clinical teachers part of and what are the contradictions in these systems? and (2) how do the contradictions manifest in the system of education?

## Methods

The study employed an interpretivist approach, acknowledging that constructions of reality are elicited through interactions between the researcher and the object under study (Bunniss and Kelly 2010). Activity theory (Engeström 1987) was applied as a theoretical framework, and qualitative data collection methods were used to gather various experiences. The research group consisted of researchers with expertise in qualitative methods and HPE (KBL, ML), clinical education (EB, AE) and educational psychology (JN). This enabled a dynamic discussion whereby the authors engaged in reflexive data collection and analysis.

### Setting

The study was conducted in a large single-faculty medical university in Sweden, offering over 20 undergraduate HPE programmes, several of which include a large amount of clinical clerkships. These clerkships take place in academic hospitals which have the threefold task of patient care, research and education. Clinical teachers are clinicians appointed to roles as educators while maintaining varying amounts of clinical work. The university has a centre for medical education and a centre for clinical education, both of which offer educational development support to clinical teachers. Each health profession discipline is separately in charge of educational development for their professionals.

### Participants

The study was part of a larger research project in which previous findings had suggested conducting educational development on a team level (Barman et al. 2016; Söderhjelm et al. 2018). Therefore, teams of clinical teachers were invited to participate in an educational development program aiming to support them in pursuing educational development projects in their own workplace. Five teams, of which three were multi-disciplinary, participated in this one-year program. Of the 17 attendees invited to join the study, one was unable to participate for personal reasons. The 16 participants represented two academic hospitals and five clinical departments in surgery and internal medicine, and comprised seven physiotherapists, four physicians, three nurses, one occupational therapist and one hospital social worker. Two were male, and the mean age was 45 years. The duration of active participation in education was: 5+ years (8 participants), 3–5 years (5 participants), 1–2 years (2 participants) and 1 year (1 participant).

As three of the authors (EB, ML, KBL) were teaching in the educational development program, the participants were invited to the study by another author (AE) and clearly informed that participation was voluntary and would bear no impact on the assessment of their performance in the program. Informed consent was obtained from all individual participants, and ethical approval was received from the Stockholm Ethical Review Board (nr: 2016/1425-31).

### Data collection

Focus groups were held in conjunction with a programme day to facilitate participation. The interview guide was developed using activity theory and was divided into three focus areas: the individual, the group and the organisation. Examples of topics were: the driving forces for pursuing education and educational development, how management and the community attended to educational matters and the structure of education in the clinical workplace.

Each of the three focus groups had five to six participants. The first group consisted of two teams working with clinical training wards, the second group of two teams from similar departments in terms of patient characteristics and tasks, though at different hospitals, and the third group of one larger team. Each focus group had a moderator (AE, KBL, ML) and an observer (JN, EB, research assistant JC) who supported the moderator in asking follow-up questions. The focus group interviews, lasting approximately one hour, were audio recorded and transcribed verbatim by AE, and all personal data were de-identified.

### Analysis

The activity systems of the academic hospital were sought by reading and re-reading transcripts (AE, EB, KBL), identifying potential systems (AE, EB, KBL), and discussing the emerging systems (all authors). The data were then interrogated for discursive manifestations of contradictions, distinguishable as dilemmas, conflicts, critical conflicts and double binds (Engeström and Sannino 2011). Dilemmas and conflicts are expressed with the linguistic cues ‘but’ and ‘no’, respectively. Critical conflicts are expressed using narrative stories, including emotional accounts, while double binds are represented by expressions of helplessness (Engeström and Sannino 2011). The linguistic cues and a careful reading of the data were used to identify manifestations of contradictions. The different kinds of manifestations were grouped, acknowledging that they overlapped and intertwined.

Next, a thematic approach (Braun and Clarke 2006) was applied as follows: coding of manifestations (AE), collating codes into themes (AE, KBL, ML), and reviewing the emerging themes (all authors). At this stage of the analysis, we also considered the mediations between elements and systems that emerged during the analysis of contradictions.

We recognise that some of the authors’ (EB, ML, KBL) involvement in the educational development program might have influenced the analysis. Therefore, we iteratively discussed the ongoing analysis until consensus was reached, continuously challenging any assumptions not supported by the data. Also, the main author (AE) kept a reflective log, documenting decisions and reflections on the ongoing analysis.

## Results

### Activity systems of the academic hospital: patient care, research and education

In keeping with activity theory, we divided the activity of the academic hospital into three activity systems directed by the objects of patient care, research and education respectively (Table 1). These activities join together in the desired outcome of the academic hospital: improved health. The clinical teachers were subjects in several or all of these three systems, yet with different roles and responsibilities. In the section below, the contradictions between these systems will be described followed by a discussion of how the contradictions, as well as mediators, manifested in the activity of education.

**Table 1:** The three activity systems of the academic hospital

	Subject	Object	Community	Tools	Rules	Division of labour	Desired outcome
System of patient care	Health care professional	Patient care	Clinical community	Medical technology and devices^a^	Always put the patient first^a^	Professions e.g. nurse, physician	Improved health
System of research	Researcher	Research	Researcher community	Research funding^a^	Only pursue ethically sound research^a^	Academic degrees e.g. professor, Ph.D.	Improved health
System of education	Clinical teacher	Education	Educator community	Educational development programs	Actively participate in educational development	Educational roles e.g. adjunct clinical teacher, supervisor	Improved health

^a^Illustrative examples

#### Contradicting objects between the systems of the academic hospital

**Fig. 2:**
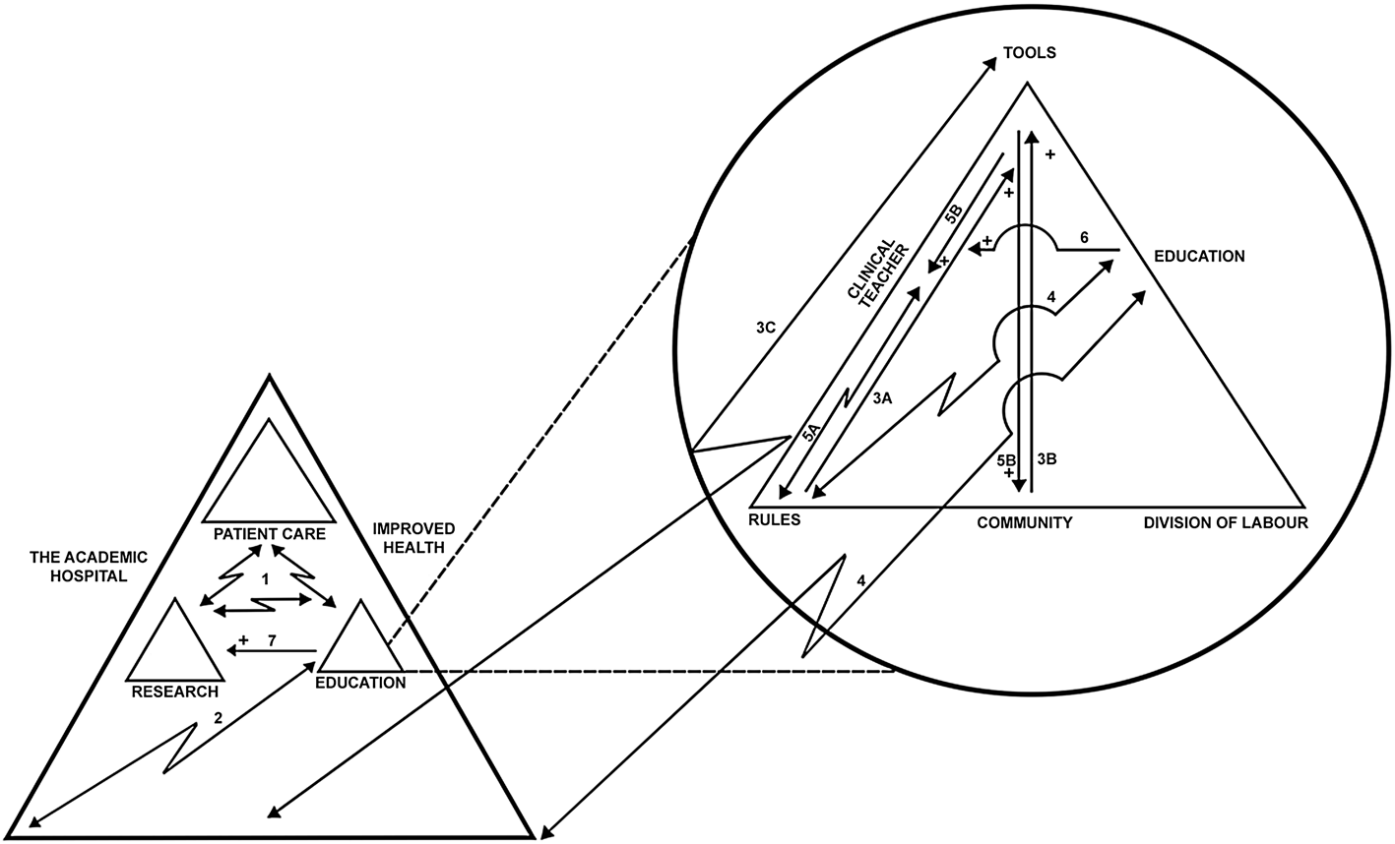
The contradictions in the activity systems of the academic hospital. Clinical teachers are part of multiple activity systems in the academic hospital between which tensions arise as their objects of patient care, research and education are misaligned, leading to some activities being prioritized over others (illustrated with different sizes of triangles). This contradiction manifests in multiple ways in the activity of education (zigzag arrows), while there were also examples of mediations within and between systems (plain arrow followed by a +). Figure key: 1. Contradicting objects between the systems of the academic hospital. 2. Misalignment between espoused and enacted values of education. 3A–C. Difficulties establishing formal discussions on education in the clinical workplace. 4. Transient educational appointments hinder educational work. 5A–B. Impeding the development of a teacher identity. 6. Education provides a creative space in routinized clinical work. 7. Complementing activities—education levers research

The three activity systems were closely interconnected as they had to work together to achieve the outcome of the academic hospital, however it was evident that contradictions arose between the systems as their objects of patient care, research and education were not aligned (Fig. 2, line #1). As time was constantly scarce, some activities were prioritized over others, manifesting the contradiction between the systems and reflecting how activities held unequal value in the work of an academic hospital.

Our department drops education as soon as it gets stressed and the pressure is on, then it’s the patient first and education last. *(Participant 1, Focus group 3)*

This contradiction left the individual teacher struggling with being part of several activity systems where time had to be divided between activities. Hence, the teachers were torn between conflicting roles and identities where they had to negotiate between engaging in education and being a colleague and clinician, and sometimes also a researcher.

We feel pressured because our colleagues don’t get the back-up they need when we need to take time for educational work and if we could just land in that and be able to take those days and half days that we need to take, then we could pursue the educational work even better, without feeling pressured like we are now. […] If you have reserved a day for educational work and someone is sitting with the schedule: ‘No, do you need the whole day for educational work?’ ‘Can you do clinic half a day?’ ‘Can you at least work some hours?’ and then it becomes so clear that if I don’t try to turn myself inside out, my colleagues will have a huge job with lots of patients and a high throughput. […] That’s the most troublesome, that there is hardly any space in the system at all. *(P5, FG 3)*

This constant negotiation between the activities of the academic hospital left the teachers with little time for educational work in general, and consequently also little time for educational development which was an activity intrinsically interconnected to the activity of education.

There is no extra time to carry out educational development work, and instead, you focus on the minimum that needs to be done with as good quality as possible. But you would like to develop the education further, and you don’t have the time for that if you don’t do it in your spare time. *(P2, FG 3)*

### Manifestations of contradictions and mediators in the activity system of education

#### Misalignment between espoused and enacted values of education

The contradiction between the conflicting objects of the academic hospital manifested in the activity of education in several ways, thus shaping the activity and its elements. One of these manifestations concerned the misalignment between the espoused values of the academic hospital (what the hospital says it values) and its enacted values (what the hospital implies it values through what it actually does).

##### Espoused values: education as an integral part of working in an academic hospital

There seemed to be espoused values and norms about education as an official obligation and integral part of working in an academic hospital and not just a responsibility limited to those with an appointed educational role. Further, the participants described how management’s approach towards education could impact the general approach towards education in the clinical community where some participants described having a management with an articulated educational focus.

It [management] has a huge impact. It’s a way of signalling that education is allowed to take space, not only as they say, but also that something happens, that they want to work with it [education]. So I think that management communicates either that they have an interest or that they don’t care, and this is reflected [among the staff]. *(P4, FG 2)*

##### Enacted values: lacking support and formal acknowledgment for education

These espoused values seemed to misalign with the enacted values of the hospital. For example, the misalignment was reflected in the lack of support and buy-in from management for educational work and as such, this misalignment shaped the rules that regulated the activity of education (Fig. 2, line #2).

I haven’t really gotten the feedback that I wanted. It’s difficult because at the same time as they say that they want student wards and want us to supervise, we don’t get the resources for it. It’s like fighting an uphill battle, of course. The university wants us to sign a contract with the clinical department, which I have done, but it’s still lying there [at the department head’s desk], I haven’t got it signed […] But of course, there is positive cheering, but sometimes it’s backbreaking. *(P6, FG 1)*

Further, some participants related that educational representatives were missing from the department’s board of directors or that management rarely took part in educational discussions. In one case, management was described as having delegated the responsibility for educational matters fully to the clinical teachers. This highlighted the lack of support and buy-in from management and reflected how the division of labour between patient care, research and education had shifted the responsibility for education to those with educational roles, while leaving educational representatives outside the forums in which decisions were made.

*P1:* Well, the feeling has always been that if you have an adjunct teacher and some clinical teachers then you as a manager don’t need to care about education. […] Because then you have people that are supposed to take care of it. […] And that’s where I feel I might have struggled a bit, trying to get them [the managers] to understand that they must also be involved in it.

*Interviewer:* How do you think they should be involved?

*P1:* Well, you should know that you have students [laughs]. No, but we’ve talked a lot about this with time, so who does the scheduling during the student period and understands how much time it actually takes with students and teaching. Now you have to try to pull through while doing your clinical job at the same time.

*P5:* Yes that was a good description. Like, [education] cannot take any time because it’s always the patient first.

*P1:* And I think it’s because they [the managers] don’t understand how it is. […] They [the managers] have like ‘well you’ll take care of that’. *(FG3)*

Another manifestation of the misalignment between the espoused and enacted values was that many teachers experienced a lack of formal acknowledgment for educational work, despite the espoused values of education as integral to academic hospitals. This lack of formal acknowledgment included for example the lack of lectureships for clinicians engaged in education and some discussed how this lack of formal acknowledgment might have led to others leaving the educator career path.

#### Difficulties establishing formal discussions on education in the clinical workplace

The participants described how educational discussions with peers also engaged in education nurtured their engagement and supported them in their teaching role, thus acting as a tool in the education activity system. These discussions partly occurred in informal arenas, where teachers and peers had educational conversations during the working day. The actual coming together in these informal gatherings was mediated by routines and facilities such as schedules and lunch habits, having educationally interested peers nearby as well as having access to meeting places where these discussions could take place (Fig. 2, line #3a).

I find it very inspiring to be in smaller groups if you know that these people are also interested in education, in a context where you yourself are situated. Educational development courses form a base but then it’s supposed to be implemented, and then you need some friends who think together ‘how can we proceed here and now?’ so that you have a team where you can inspire and support and develop together. *(P4, FG 2)*

Further, educational discussions were also mediated by access to formal arenas such as scheduled days assigned for education, where clinical teachers would meet with other teachers in the hospital as well as with representatives from the university to discuss educational matters. However, these formal arenas were often off-site and initiated by the university, as well as being quite infrequent (usually one or two times a year). In some workplaces, champions in the educator community had made efforts to formalize educational discussions at a departmental level (Fig. 2, line #3b). In other cases, participants experienced few or no opportunities for formal or structured educational discussions in the clinical community, with education apparently missing from the common agenda of the community in the academic hospital (Fig. 2, line #3c). This indicated how different communities either allowed or disallowed certain discussions in the workplace and thus shaped the way in which teachers gathered to discuss education.

In the workplace, then it’s mostly me and the professor [the former clinical teacher] that tries to carry out different things and we don’t have any real structure or regularity for meetings actually, it’s once a semester but without, what shall I say, any clear plan. *(P4, FG2)*

#### Transient educational appointments hinder educational work

The division of labour in the academic hospital shaped the rules of the activity of education, and appeared to impede teachers in their work towards their object of education (Fig. 2, line #4). Partly, this had to do with how educational roles were staffed. While some participants had specifically applied for a teaching position, many of them had not intentionally sought the teaching position as part of a career trajectory. Instead, the appointment of clinical teachers was often carried out by allocating educational roles to members of the clinical community. As an example of this, one department was planning for residents to rotate as clinical teacher as part of their residency.

I was asked to do it, or our professor and our head of department asked me if I was interested in being a clinical teacher […] and by then I didn’t know what it entailed, but it felt, or it sounded fun. And I said yes without having had the time to reflect on it so it was during the process that I also became interested in education. *(P1, FG 1)*

Moreover, the employment as teacher was often limited to one or two years which meant that the role often shifted between individuals. This was experienced as limiting the opportunities for educational work and educational development in a longer-term perspective as it hindered continuity.

*P3:* We have our assignments for two years, and it’s a pretty high turnover, and as we don’t have any education [as teachers], it takes some time to acquaint oneself with the position, and then it’s time to quit really.

[…]

*P4:* And that makes it difficult if you want to carry out some [educational] development work because then, all of a sudden, there’s a new co-worker who needs to be introduced. *(FG 3)*

#### Impeding the development of a teacher identity

The participants did not seem to identify fully with the role as educator as they lacked formal training and knowledge within education.

But you just realise that there is much more than the tiny, tiny bit that I know because we don’t have any educational training; we’re not teachers. *(P3, FG 3)*

As such, it seemed as if one of the rules or norms of the education system was that formal training in education was required to become a legitimate member of the educator community and to identify oneself with the educator role (Fig. 2, line #5a). Therefore, the participants partly sought out educational development activities as a tool offering formal training and thus, educational development could mediate the establishment of an identity as teacher and the sense of belonging to the community of educators (Fig. 2, line #5b).

I’m not really here to change so much, I’m here to learn because I’m so new in everything, teaching and learning is new […] So this [the educational development program] is a way of entering it [education] as a science or something you work with. *(P2, FG 1)*

#### Education mediates a creative space in routinized clinical work

The activity system of education, in which education was the generalized object on the system level, provided the participants with several potential outcomes on an individual level. Partly, these outcomes related to a personal interest in teaching where education mediated personal and professional development (Fig. 2, line #6). Also, many of the teachers were unsatisfied with the space for creativity within the activity of patient care and felt insufficiently stimulated by clinical work which had become routine. Therefore, the educational role attracted as it mediated a creative space with a freedom to manoeuvre and to be self-directed, and entailed a qualitatively different way of working than traditional clinical work.

It’s the freedom. There is so much that is controlled in an academic hospital, and in this role, I get an outlet for all my ideas, […] I mean, it’s fun, things happen, so it’s where my freedom is. (*P3, FG 3)*

#### Complementing activities—education levers research

As described above, the teachers experienced a lack of formal acknowledgement for educational work. However, there were efforts in some departments to remunerate teachers for their work, for example by offering time and funding for research.

The clinical teacher has 33% of his or her time reserved for education. […] In our department, the teaching role generates an additional 33% time for research […] money comes from a specific budget that funds this 33% [time] for research. *(P1, FG 1)*

Also, as education was perceived as a qualification within an academic career, engaging in education offered further leverage in the activity of research (Fig. 2, line #7).

Another thing that makes people motivated to teach in our department is that we have a lot of research. And teaching, especially on that level [as clinical teacher], teaching medical students, is very meriting in research, so there are lots of people [in the department] who want to teach because of that. *(P1, FG 1)*

## Discussion

In this study, we employed an activity theory perspective to explore how clinical teachers’ engagement in educational development was affected by the systems they act within. We found that the clinical teachers were part of three interacting activity systems of the academic hospital: the systems of patient care, research and education. Contradictions arose between these three activities as they had differing objects, resulting in some activities being prioritised over others. This has multiple implications for education and educational development, as teachers’ priorities seem to be negotiated in interaction with the priorities of the academic hospital.

Our findings suggest that education is a marginalised activity of the academic hospital where the community allows or disallows certain activities. In our study, this manifested in a number of examples discussed by the participating clinical teachers, including the mismatch between the espoused and enacted values of the academic hospital, the limited opportunities for formal educational discussions in the clinical workplace, the lack of educational representatives in the forums in which decisions were made, and the constant negotiation between education and patient care imposed on the teachers by scheduling and workloads. Thus, the competing objects of the academic hospital meant that the teachers were constantly pulled in different directions.

This tension between education and other tasks of an organisation, whether it is patient care in hospitals or research in universities, has been described as impeding teachers’ possibilities to carry out educational change and educational development (Cantillon et al. 2016; Jääskelä et al. 2017; Stenfors-Hayes et al. 2010; Zibrowski et al. 2008). It has been suggested that this relates to leadership and culture in an organisation (Jääskelä et al. 2017; O’Sullivan and Irby 2011), where teachers’ agency and their opportunities to develop educational practices is decreased in hospitals where education has low status (Cantillon et al. 2016). As such, teachers’ priorities seem to closely interact with the values and practices of the academic hospital, and our study suggest that the contradictions between education and patient care may restrict teachers’ opportunities to pursue educational development and change.

In the light of the present study illustrating how clinical teachers are pulled between activities with contradicting objects in the academic hospital, the burning question remains how to enable these teachers to engage in educational development. Some possible ways to address this complex question were those instances of mediation suggested by this study, one of which was educational discussions. These discussions emerged as a crucial tool in the activity of education as they supported teachers in their educational role and enabled the creation of communities of educators. This appears especially important considering previous findings where communities of teachers have been found to collectively advance teachers understanding of education, with the workplace being highlighted as a valuable source of learning (Jääskelä et al. 2017; Knight et al. 2006; O’Sullivan and Irby 2011; Roxå and Mårtensson 2009). Therefore, creating arenas allowing educational discussions in the workplace emerges as an important task for educational developers and managers in academic hospitals, thus promoting and sustaining communities of educators and enabling learning and educational development in the clinical workplace.

While the above underscores the importance of workplace learning and communities of educators for educational development, some of the participants in this study did not percieve themselves as teachers as they lacked formal training in education. Accordingly, one of the ‘rules’ of the education system seemed to be that one needs formal training to become a legitimate member of the educator community. This relates to the incentive to engage in educational development activities to increase knowledge and skills within teaching as described here and elsewhere (Bouwma-Gearhart 2012; Steinert et al. 2010; Stenfors-Hayes et al. 2010). Another factor that might have limited participants’ opportunities to develop teacher identities and become legitimate members of the educator community was the transience of educational roles, which were often allocated rather than sought out. Previous research suggests that teacher identity is negotiated in relation to the culture of the workplace (Cantillon et al. 2016), which could explain why the values and practices of the academic hospital in our study might have further impeded the participants’ sense of being teachers. Our findings suggest that educational development have the potential to function as a tool mediating the formation of clinicians’ teacher identity and their legitimate membership in the educator community. As such, educational development may have a central role to play in overcoming some of the contradicting values experienced in the workplace. We therefore agree with previous suggestions to focus on teacher identity in educational development (Steinert and MacDonald 2015; Stone et al. 2002).

The findings of the present study have implications for educational development in suggesting that the systems clinical teachers participate in are important in determining whether or not they have opportunities to develop their teaching practice, to conduct educational change in the clinical workplace, and to develop their identities as teachers. For these reasons, we believe that educational developers should shift focus from individual teachers to also developing activity systems by addressing the inherent rules, division of labour and communities. We therefore concur with others and regard it as imperative that the context in which clinical teachers work is taken into account in future educational development activities (Jääskelä et al. 2017; O’Sullivan and Irby 2011). Moreover, we suggest that those with power in the clinical workplace, such as management, also need to take part in educational development activities as it would enable us to establish educational leadership in the workplace (Bolander Laksov and Tomson 2017) and to create clinical workplaces in which the advancement of teaching is recognised and valued (Knight et al. 2006). Also, we strongly support the inclusion of clinical teachers or other educational representatives in the decision-making bodies of clinical departments, especially considering the finding that this was not always the case. The above interventions taken together would not only promote an educational perspective in decision-making but would hopefully also help us to better align the activities of the academic hospital, thus supporting clinical teachers in engaging in educational development.

Further, the findings suggest expanding the possibilities to pursue a career as clinical educator. This would require distinct educational career paths where education is meriting, starting with the hiring practices in education where we should move away from allocating or appointing educational roles to clinicians. Instead, these appointments must be made attractive by having a clear and focused job description, and also through the establishment of reward structures. As pointed out in the findings, one such reward structure could be to create educational lectureships or similar roles, as this would contribute to making the educational career path for clinicians visible and appealing.

Lastly, due to the contradictions between the activity systems of the academic hospital as highlighted in this study, we believe that simply adding more time is insufficient in supporting clinical teachers engagement in educational development, risking that time is again distributed unevenly between patient care, research and education. Instead, one solution could be to establish a system in which educational development is required of clinical teachers, with time earmarked for such activities whether it be attending workshops and courses, partaking in educational conferences or pursuing educational development in the own workplace. This would call for a continuous dialogue between the hospital and faculty development units in the university, where the support is tailored to the needs of the individual clinical teacher, as well as to the activity system of education.

### Limitations and future research

Given that this study only included one faculty of medicine, with participants drawn from one educational development program, the transferability of findings might be limited. However, several professions and clinical environments were represented, indicating diverse backgrounds and various experiences of engaging in educational development.

Interestingly, the findings suggest education to lever the activity of research by providing merits, funding and time. We do not know if the effect goes also in the opposite direction, or if this might serve as a way of aligning these activities, why this could merit further exploration. Also, while this study included participants from different health professions, it did not focus on differences between professions regarding the opportunities for educational development, a question requiring further studies.

Lastly, while this study offers insight into the engagement of teachers who do participate in formal educational development, there are still unanswered questions about teachers who do not participate in such activities and the possible barriers that they encounter.

## Conclusion

In this study, we employed an activity theory perspective to explore how clinical teachers’ engagement in educational development was affected by the systems they act within. Our results situate clinical teachers as part of an academic hospital where the systems of patient care, research and education co-exist and interact, and suggest that contradictions arise between these systems as their objects are misaligned. Further, the findings suggest that the priorities of clinical teachers are negotiated in interaction with the priorities of the academic hospital, where the activity of education appears to be less valued and prioritised, thus limiting the opportunities for educational development. Therefore, while educational development activities are important in supporting and developing individual clinical teachers, we must also focus on developing systems where the advancement of education is promoted and valued, thus mediating clinical teachers’ engagement in educational development. Accordingly, we would like to suggest including educational representatives in decision-making bodies in the hospital, including management and leaders of academic hospitals in educational development, as well as supporting the creation of communities of educators to better enable educational development in the workplace. Similarly, changing the way in which teaching is valued and rewarded, for example by establishing distinct educational career paths with clear reward structures, might hopefully serve to support clinical teachers and their pursuit of improving their teaching practice.
